# Mercury Levels and Their Determinants in Women of Reproductive Age in the Brazilian Amazon: A Cross-Sectional Study

**DOI:** 10.1007/s00128-026-04320-0

**Published:** 2026-08-20

**Authors:** Leonardo Breno do Nascimento de Aviz, Renan Rocha Granato, Fabiana Costa Cardoso, Carolina Fonte de Aviz, Laura dos Santos Barros, Brena Karoline Ataide Furtado, Rayanne Vieira da Silva, Márcia Cristina Freitas da Silva, Saul Rassy Carneiro, Maria da Conceição Nascimento Pinheiro

**Affiliations:** 1 Tropical Medicine Center, Federal University of Pará, Belém, 66005-240 Brazil; 2 Faculty of Medicine, Institute of Health Sciences, Belém, 66005-240 Brazil; 3 Institute of Biological Sciences, Federal University of Pará, Belém, 66075-110 Brazil; 4 Postgraduate Program, Tropical Medicine Center, Federal University of Pará, Belém, 66075-110 Brazil

**Keywords:** Mercury, Environmental exposure, Fish consumption, Women’s health

## Abstract

Mercury exposure remains a public health concern in Amazonian populations. This study assessed hair total mercury levels and associated determinants in 205 women of reproductive age from riverine communities in the Lower Tocantins and Xingu regions, Pará,Brazil. Hair Hg concentrations were measured using cold vapor atomic absorption spectrometry. Multivariable linear regression was used to identify predictors of log-transformed mercury total levels. Median concentration was 0.332 µg/g, with 85.4% below 1 µg/g. Women from the Lower Tocantins had significantly higher levels than those from the Xingu (*p* < 0.001). Region (β = −0.516; *p* < 0.001) and high fish consumption (> 4 times/week; β = 0.207; *p* = 0.024) were significant predictors. Mercury levels were low, but regional differences and high fish consumption frequency were associated with exposure, highlighting the need for targeted biomonitoring strategies.

## Background

Environmental exposure to chemical contaminants, particularly heavy metals, represents a major global public health concern. Mercury (Hg) is among the top ten chemicals of major public health concern according to international organizations (Ray et al. 2025). Hg persists in the environment due to its resistance to degradation and undergoes complex biogeochemical transformations, including microbial methylation, which produces methylmercury (MeHg), its most toxic and bioaccumulative form (Ray et al. 2025; Pinheiro et al. 2025) The Brazilian Amazon has been historically impacted by Hg contamination (Lourenço et al. 2026). Artisanal and small-scale gold mining represents a major anthropogenic source of Hg emissions in Latin America, contributing to environmental and human exposure for over a century in this region (Han et al. 2023). Consequently, populations that rely on fish as their primary source of animal protein may experience higher levels of Hg exposure and associated toxic effects (Nyholt et al. 2022).

The Lower Tocantins and Xingu regions differ in aspects relevant to mercury exposure. The Lower Tocantins Basin has a longer history of artisanal and small-scale gold mining and higher levels of anthropogenic mercury input into the aquatic environment (Wasserman et al. 2025; Lourenço et al. 2026). In contrast, the Xingu region, although not exempt from mining influence, presents distinct hydrological and geochemical characteristics that may modulate mercury methylation and bioaccumulation in fish (Pestana et al. 2019; Lino et al. 2019)**.**

In women, Hg acts primarily as a neurotoxic agent and has deleterious effects on the reproductive system (Dutta et al. 2022; Marconi et al. 2026). MeHg readily crosses the blood–brain and placental barriers, reaching the developing fetal nervous system and increasing the risk of adverse outcomes such as miscarriage, low birth weight, and preterm birth (Clarkson 1993; Zhang et al. 2023). It has also been associated with menstrual and hormonal disturbances, infertility or subfertility, and impaired ovarian function (Dutta et al. 2022; Marconi et al. 2026).

This study aimed to assess hair mercury levels and identify sociodemographic, environmental, and behavioral determinants of exposure among women of reproductive age living in riverine communities in the Lower Tocantins and Xingu regions of the Brazilian Amazon. We hypothesized that mercury levels would differ between regions and would be positively associated with fish consumption. 

## Methods and Materials

This was a cross-sectional observational study conducted between January 2023 and March 2024. The study followed the guidelines established by Strengthening the Reporting of Observational Studies in Epidemiology (STROBE) (von Elm et al. 2008). The study population consisted of women residing in riverine communities in the Lower Tocantins and Xingu regions, Pará State, Brazil (Supplementary Material). Participants were selected using a non-probabilistic consecutive sampling approach, including all eligible and available women during the data collection period according to predefined inclusion and exclusion criteria.

Inclusion criteria were: age between 18 and 55 years, residence in the study communities for more than three years, and provision of written informed consent. Exclusion criteria were: current pregnancy, active infectious disease at the time of collection, cognitive impairment precluding questionnaire completion, and severe acute or chronic conditions that could compromise participation. Data were collected through household visits using a structured questionnaire that included sociodemographic characteristics, housing conditions, lifestyle habits, environmental exposure, and reproductive history, all self-reported by the participants. The dependent variable was total hair mercury (HgT) concentration (µg/g), which was log10-transformed (log10Hg) for inferential analyses.

Mercury exposure was assessed through hair sample analysis. Samples were collected from the occipital region using pre-cleaned stainless-steel scissors, cutting a small lock of hair close to the scalp. Samples were washed prior to analysis, followed by acid digestion. Total mercury concentration was determined using cold vapor atomic absorption spectrometry. Quality assurance and quality control included calibration with external mercury standards (10, 20, and 50 ng), procedural and reagent blanks, and repeated measurements. Calibration curves showed good linearity (r = 0.9942), and analytical performance was considered satisfactory based on instrument stability and negligible contamination throughout the analyses.

Descriptive statistics were used to summarize the characteristics of the study population. Comparisons of total hair mercury (HgT) levels between regions were performed using the Mann–Whitney U test based on non-transformed data. A two-tailed *p*-value < 0.05 was considered statistically significant. A multivariable linear regression model was constructed to assess the association between sociodemographic, reproductive, and environmental factors and log10-transformed hair mercury levels (log10Hg). Descriptive analyses of log10Hg concentrations were also conducted according to categories of fish consumption, both for the overall sample and stratified by region.

## Results

A total of 205 women were included in the study. Participants had a median age of 27 years (IQR: 21–37) and a median body weight of 67 kg (IQR: 58.6–77). Most participants self-identified as non-white (88.8%), had ≥ 9 years of education (61%), and reported a household income below one minimum wage (68.8%).Additional sociodemographic and lifestyle characteristics are presented in Table 1.

**Table 1:** Sociodemographic, lifestyle, and clinical characteristics of the study (N = 205)

Variable	Lower Tocantins (n=100) (%)	Xingu (n=105) (%)	Total (n=205) (%)
*Race*			
Non-white	88 (88.0)	94 (89.5)	182 (88.8)
White	12 (12.0)	11 (10.5)	23 (11.2)
*Education (years)*			
< 9 years	43 (43.0)	37 (35.2)	80 (39.0)
≥ 9 years	57 (57.0)	68 (64.8)	125 (61.0)
*Household income*			
< 1 minimum wage	74 (74.0)	67 (63.8)	141 (68.8)
1–2 minimum wages	25 (25.0)	38 (36.2)	63 (30.7)
> 2 minimum wages	1 (1.0)	0 (0.0)	1 (0.5)
*Smoking*			
Yes	3 (3.0)	2 (1.9)	5 (2.4)
No	97 (97.0)	103 (98.1)	200 (97.6)
*Smoking history*			
Yes	15 (15.0)	17 (16.2)	32 (15.6)
No	85 (85.0)	87 (82.9)	172 (83.9)
*Alcohol consumption*			
Sometimes	47 (47.0)	35 (33.3)	82 (40.0)
Often	6 (6.0)	6 (5.7)	12 (5.9)
Never	47 (47.0)	64 (61.0)	111 (54.1)
*BMI category			
Underweight	7 (7.0)	1 (1.0)	8 (3.9)
Normal weight	24 (24.0)	35 (33.3)	59 (28.8)
Overweight	43 (43.0)	39 (37.1)	82 (40.0)
Obesity	26 (26.0)	30 (28.6)	56 (27.3)
*Fish consumption*			
Low (< 2/week)	29 (29.0)	80 (76.2)	109 (53.2
Moderate (2–4/week)	53 (53.0)	17 (16.2)	70 (34.1)
High (> 4/week)	18 (18.0)	8 (7.6)	26 (12.7)
*Hair dye use*			
Yes	56 (56.0)	59 (56.2)	115 (56.1)
No	44 (44.0)	46 (43.8)	90 (43.9)
*Chemical exposure*			
Yes	11 (11.0)	8 (7.6)	19 (9.3)
No	89 (89.0)	97 (92.4)	186 (90.7)
*Mercury vapor exposure*			
Yes	5 (5.0)	1 (1.0)	6 (2.9)
No	95 (95.0)	104 (99.0)	199 (97.1)

^*^BMI = Body mass index. Values are presented as median (interquartile range) and minimum–maximum for continuous variables and n (%) for categorical variables

Regarding reproductive health, participants had a median of 2 children (IQR: 1–3). Most women had 1–2 children (55.1%), and 42.4% reported contraceptive use. Detailed reproductive characteristics are shown in Table 2.

**Table 2:** Reproductive characteristics of the study participants (n = 205)

Variable	Category	Values	
		Median (IQR)*	
Number of children		2 (1–3); min–max: 0–13	
Number of deliveries		2 (1–3); min–max: 0–16	
Number of abortions		0 (0–1); min–max: 0–4	
		**N (205)**	**% (100)**
PARITY	0 Children	15	7.3
	1–2 Children	113	55.1
	3–4 Children	57	27.8
	≥5 Children	20	9.8
CONTRACEPTIVE USE	Yes	87	42.4
	No	118	57.6
AGE AT MENARCHE	<12 years	56	27.3
	12–18 years	143	69.8
	>18 years	6	2.9
AGE AT SEXUAL DEBUT	<12 years	10	4.9
	12–18 years	148	72.2
	>18 years	47	22.9
CONDOM USE	Always	30	14.7
	Sometimes	88	43.1
	Never	86	42.2
NEONATAL HEALTH PROBLEMS	Yes	26	12.7
	No	164	70
	No children	15	7.3
DISEASES DURING PREGNANCY	Yes	10	4.9
	No	180	87.8
	No children	15	7.3

Values are presented as median (interquartile range) and minimum–maximum for continuous variables and n (%) for categorical variables

**Fig. 1:**
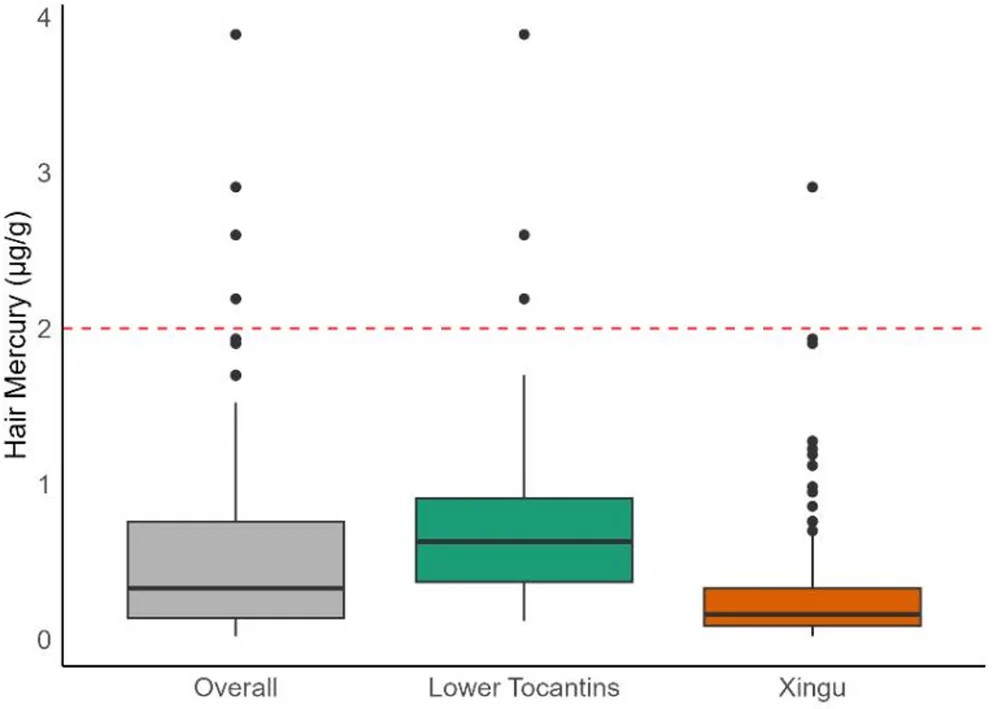
Distribution of HgT mercury concentrations in Amazonian regions. Overall distribution of HgT levels in the study population and distribution of HgT levels according to region of residence. The dashed red line indicates the reference value of 2 µg/g for HgT mercury concentration

Median hair HgT concentration in the overall sample was 0.332 µg/g (IQR: 0.14–0.76). Women from the Lower Tocantins region had significantly higher Hg levels compared to those from the Xingu region (0.632 vs 0.163 µg/g; *p* < 0.001) (Fig. 1).

In multivariable analysis region was strongly associated with Hg levels. Women from the Xingu region had significantly lower log10Hg levels compared to those from the Lower Tocantins (β = −0.516; 95% CI −0.665 to −0.366; *p* < 0.001). Fish consumption was also associated with mercury exposure. Compared to women consuming fish less than twice per week, those consuming fish more than four times per week had significantly higher mercury levels (β = 0.207; 95% CI 0.027–0.388; *p* = 0.024), while no statistically significant association was observed for those consuming fish 2–4 times per week (β = 0.120; 95% CI −0.017 to 0.257; *p* = 0.085) (Table 3).

**Table 3:** Multiple linear regression analysis associated with hair HgT levels (logHg)

Variable	β	95% CI	*p*-value
*Region*			
Xingu vs Lower Tocantins	−0.516	−0.665; − 0.366	**<0.001**
Age (years)	0.000	−0.006; 0.006	0.973
*Education level*			
≥ 9 years vs < 9 years	−0.033	−0.153; 0.088	0.595
*Household income*			
1–2 minimum wages vs < 1	−0.054	−0.182; 0.075	0.409
> 2 minimum wages vs < 1	−0.452	−1.237, 0.333	0.258
*Fish consumption*			
2–4 times/week vs < 2	0.120	−0.017; 0.257	0.085
> 4 times/week vs < 2	0.207	0.027; 0.388	**0.024**
*Contact with chemical products*			
Yes vs No	0.153	−0.059; 0.364	0.156
*Exposure to mercury vapor*			
Yes vs No	−0.191	−0.557; 0.174	0.303
Number of children	−0.011	−0.039; 0.018	0.450
Number of abortions	−0.038	−0.128; 0.052	0.406
*Neonatal health problems*			
Yes vs No	−0.036	−0.184; 0.113	0.634
*Age at menarche*			
≥ 12 years vs < 12 years	0.020	−0.093; 0.133	0.729

Coefficients represent changes in log10-transformed hair HgT levels. Confidence intervals (95% CI) were calculated for all estimates. Reference categories were: Lower Tocantins for region; < 9 years for education level; < 1 minimum wage for household income; < 2 times/week for fish consumption; and absence of exposure or condition for binary variables

**Fig. 2:**
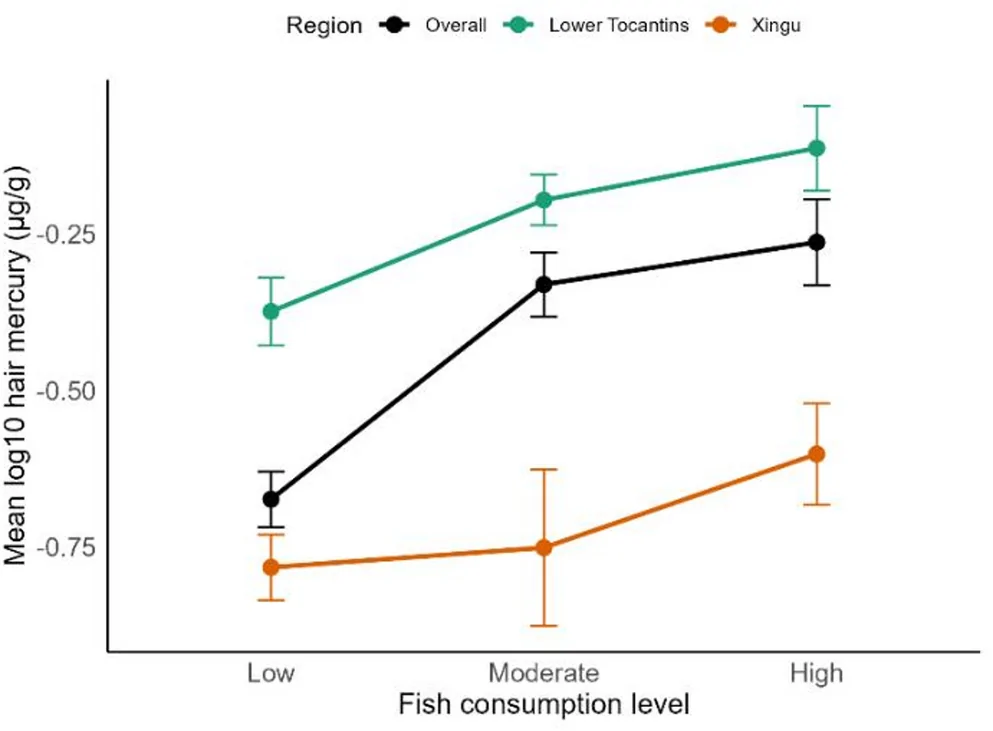
Dose–response relationship between fish consumption and hair mercury levels. Note: Points represent mean values and error bars indicate standard errors

A clear dose–response patter was observed between fish consumption and mercury levels (Fig. 2). Mercury concentrations increased with higher fish consumption in both regions, with consistently higher levels observed in the Lower Tocantins across all consumption categories.

## Discussion

This study assessed hair HgT levels and their determinants in 205 women of reproductive age living in riverine communities in the Brazilian Amazon. Overall, HgT concentrations were lower than those reported for other riverine and Indigenous populations across the region. Most participants (85.4%) had HgT concentrations below 1 µg/g, and only 2.0% exceeded the reference value of 2 µg/g. In contrast, studies from other Amazonian regions have reported substantially higher concentrations, including mean HgT levels of 7.82 µg/g along the Madeira River (Silva et al. 2026), 6.5 µg/g in the Tapajós region during the dry season(Esplugas et al. 2025) and values ranging from 0.8 to 83.89 µg/g among Indigenous populations(Pinheiro et al. 2025). Together, these findings illustrate the marked regional variability in Hg exposure across the Amazon and reinforce the need for continuous, region-specific biomonitoring.

The comparatively lower HgT levels observed in this study may be explained, at least in part, by differences in fish consumption and regional environmental characteristics. More than half of the participants (52.2%) reported eating fish fewer than twice per week, contrasting with the daily consumption commonly observed in other Amazonian communities, where fish represents the main source of methylmercury exposure(Dolbec et al. 2001; Silva et al. 2026).

This pattern may reflect the ongoing dietary transition in the Amazon, characterized by reduced consumption of traditional foods and greater reliance on processed products (Oestreicher et al. 2020). In addition, the regions included in this study may be less directly impacted by artisanal gold mining compared to river basins such as the Tapajós and Madeira, which have historically experienced more intense mining activity (Souza-Araujo et al. 2022).

The positive association between fish consumption frequency and hair HgT levels observed in this study is consistent with the existing literature on Hg exposure in Amazonian populations. MeHg, the most toxic and bioaccumulative form of Hg, accounts for approximately 90% of HgT in hair and is strongly associated with fish consumption frequency (Esplugas et al. 2025). Studies conducted in the Tapajós region have demonstrated that individuals who consume fish daily present significantly higher hair Hg levels compared to those who consume fish only a few times per week (Dolbec et al. 2001). In the present study, a clear dose–response relationship was observed in both regions. However, this gradient was more pronounced in the Lower Tocantins, where baseline environmental contamination levels appear to be higher.

The significant difference in Hg levels between the Lower Tocantins and Xingu regions is a important finding. Regional variability in Hg exposure has been widely documented across the Amazon and may be attributed to multiple factors, including proximity to artisanal gold mining areas, local geochemical characteristics, and differences in fish species consumption that influence Hg methylation (Kasper et al. 2017; Souza-Araujo et al. 2022). The relative contribution of these factors varies geographically, which may explain the differences observed between the regions evaluated in this study.

Assessing Hg exposure in women of reproductive age is particularly important given the ability of MeHg to cross the placental barrier and adversely affect fetal neurodevelopment. Studies in Munduruku Indigenous communities have reported MeHg levels above 6.0 µg/g in most women of reproductive age, with mean concentrations reaching 12.67 µg/g in the most affected areas. Basta et al. (2021), Kempton et al. (2021). In contrast, the low prevalence of elevated Hg exposure observed in this study is a favorable finding. Nevertheless, fish consumption and region of residence were important determinants of exposure and may help guide targeted public health strategies for higher-risk populations in the Amazon.

Although most participants in this study presented HgT levels within ranges considered safe, the association between fish consumption frequency and mercury levels was statistically significant only among women consuming fish more than four times per week. For lower consumption categories, no significant association was observed in this population. Therefore, dietary recommendations should be approached with caution and grounded in local epidemiological data rather than generalized guidance. Region of residence and biomonitoring strategies adapted to local contexts remain key priorities for public health in the Amazon (Hacon et al. 2020; Silva et al. 2026). Additionally, the observed regional differences in HgT levels between the Lower Tocantins and Xingu highlight the importance of adapting biomonitoring strategies to local contexts, taking into account variability in contamination sources and dietary patterns across regions.

## Conclusion

Therefore, women of reproductive age from riverine communities in the Lower Tocantins and Xingu regions presented generally low hair HgT levels, with most participants below 1 µg/g. Region of residence and fish consumption frequency were the main determinants of exposure, particularly among women from the Lower Tocantins, indicating a dose–response relationship between fish intake and mercury levels. These findings suggest that non-Indigenous riverine communities located in areas less impacted by artisanal mining may exhibit distinct exposure patterns compared to other Amazonian regions. The identification of modifiable factors, such as fish consumption, provides important insights for developing culturally appropriate public health strategies that balance the nutritional benefits of fish intake with the reduction of Hg exposure.

## References

[CR1] Basta PC, Viana PVdeS, Vasconcellos ACSde et al (2021) Mercury exposure in Munduruku Indigenous communities from Brazilian Amazon: methodological background and an overview of the principal results. Int J Environ Res Public Health 18:9222. 10.3390/ijerph18179222 34501811 PMC8430525

[CR2] Clarkson TW (1993) Mercury: major issues in environmental health. Environ Health Perspect 100:31–38. 10.1289/ehp.9310031 8354179 PMC1519577

[CR3] Dolbec J, Mergler D, Larribe F et al (2001) Sequential analysis of hair mercury levels in relation to fish diet of an Amazonian population, Brazil. Sci Total Environ 271:87–97. 10.1016/S0048-9697(00)00835-4 11346043

[CR4] Dutta S, Gorain B, Choudhury H et al (2022) Environmental and occupational exposure of metals and female reproductive health. Environ Sci Pollut Res 29:62067–62092. 10.1007/s11356-021-16581-9 34558053

[CR5] Esplugas J, Neves Cezarette G, Tessier E et al (2025) Dynamics of mercury speciation and selenium levels in hair under seasonal and dietary influences in an Amazonian riverside population. J Hazard Mater 495:138732. 10.1016/j.jhazmat.2025.138732 40479973

[CR6] Hacon SdeS, Oliveira-da-Costa M, Gama CdeS et al (2020) Mercury exposure through fish consumption in traditional communities in the Brazilian northern Amazon. Int J Environ Res Public Health 17:5269. 10.3390/ijerph17155269 32707799 PMC7432107

[CR7] Han Y, Jiang Y, Xiong X et al (2023) Mercury biomagnification at higher rates than the global average in aquatic ecosystems of the Qinghai-Tibet Plateau. J Hazard Mater 453:131408. 10.1016/j.jhazmat.2023.131408 37080022

[CR8] Kasper D, Forsberg BR, Amaral JHF et al (2017) Methylmercury modulation in Amazon rivers linked to basin characteristics and seasonal flood-pulse. Environ Sci Technol 51:14182–14191. 10.1021/acs.est.7b04374 29172464

[CR9] Kempton JW, Périssé ARS, Hofer CB et al (2021) An assessment of health outcomes and methylmercury exposure in Munduruku Indigenous women of childbearing age and their children under 2 years old. Int J Environ Res Public Health 18:10091. 10.3390/ijerph181910091 34639393 PMC8508331

[CR11] Lino AS, Kasper D, Guida YS et al (2019) Total and methyl mercury distribution in water, sediment, plankton and fish along the Tapajós River basin in the Brazilian Amazon. Chemosphere 235:690–700. 10.1016/j.chemosphere.2019.06.212 31279119

[CR12] Lourenço MdaSTA, Debruyne D, Di Maggio RM et al (2026) Mercury contamination in Brazil related to (historical) artisanal and small-scale gold mining activity: a narrative review with quantitative synthesis. Environ Geochem Health 48:45. 10.1007/s10653-025-02929-4 41389181

[CR13] Marconi G, Di Resta C, Naclerio A et al (2026) Heavy metals and human reproductive toxicity: mechanisms, pregnancy outcomes, and mitigation strategies. Reprod Toxicol 139:109104. 10.1016/j.reprotox.2025.109104 41203213

[CR14] Nyholt K, Jardine TD, Villamarín F et al (2022) High rates of mercury biomagnification in fish from Amazonian floodplain-lake food webs. Sci Total Environ 833:155161. 10.1016/j.scitotenv.2022.155161 35421468

[CR15] Oestreicher JS, do Amaral DP, Passos CJS et al (2020) Rural development and shifts in household dietary practices from 1999 to 2010 in the Tapajós River region, Brazilian Amazon: empirical evidence from dietary surveys. Glob Health 16:36. 10.1186/s12992-020-00564-5 PMC7178734 32321543

[CR16] Pestana IA, Almeida MG, Bastos WR, Souza CMM (2019) Total Hg and methylmercury dynamics in a river-floodplain system in the Western Amazon: influence of seasonality, organic matter and physical and chemical parameters. Sci Total Environ 656:388–399. 10.1016/j.scitotenv.2018.11.388 30513429

[CR17] Pinheiro MCN, Cardoso FC, Aviz LBN et al (2025) Mercury exposure and health effects in Indigenous people from the Brazilian Amazon: literature scoping review. Int J Environ Res Public Health 22:1159. 10.3390/ijerph22081159 40869747 PMC12386174

[CR18] Ray S, Vashishth R, Mukherjee AG et al (2025) Mercury in the environment: biogeochemical transformation, ecological impacts, human risks, and remediation strategies. Chemosphere 381:144471. 10.1016/j.chemosphere.2025.144471 40367742

[CR19] Silva FXda, Monteiro LC, Cabral CdaS et al (2026) Temporal changes and sociodemographic determinants of mercury exposure in Madeira River communities, Brazilian Amazon. Environ Pollut 398:128115. 10.1016/j.envpol.2026.128115 41974408

[CR20] Souza-Araujo J, Andrades R, Hauser-Davis RA et al (2022) Before the dam: a fish-mercury contamination baseline survey at the Xingu River, Amazon Basin before the Belo Monte Dam. Bull Environ ContamToxicol 108:861–866. 10.1007/s00128-021-03371-9 34524505

[CR21] von Elm E, Altman DG, Egger M et al (2008) The strengthening the reporting of observational studies in epidemiology (STROBE) statement: guidelines for reporting observational studies. J Clin Epidemiol 61:344–349. 10.1016/j.jclinepi.2007.11.008 18313558

[CR23] Wasserman JC, Balbueno R, Fernandez APP, Hacon SS (2025) Goldmining and ecosystem disruption in the Amazon: impacts on vulnerable communities and environmental restoration. Chemosphere 385:144582 10.1016/j.chemosphere.2025.144582 40737828

[CR22] Zhang T, Wang X, Luo Z-C et al (2023) Maternal blood concentrations of toxic metal(loid)s and trace elements from preconception to pregnancy and transplacental passage to fetuses. Ecotoxicol Environ Saf 264:115394. 10.1016/j.ecoenv.2023.115394 37625333

